# A Novel Mutation in *ABCA1* Gene Causing Tangier Disease in an Italian Family with Uncommon Neurological Presentation

**DOI:** 10.3389/fneur.2016.00185

**Published:** 2016-11-02

**Authors:** Marco Ceccanti, Chiara Cambieri, Vittorio Frasca, Emanuela Onesti, Antonella Biasiotta, Carla Giordano, Sabina M. Bruno, Giancarlo Testino, Marco Lucarelli, Marcello Arca, Maurizio Inghilleri

**Affiliations:** ^1^Department of Neurology and Psychiatry, Sapienza University, Rome, Italy; ^2^Department of Radiological, Oncological and Anatomo-Pathological Sciences, Sapienza University, Rome, Italy; ^3^Department of Cellular Biotechnologies and Hematology, Sapienza University, Rome, Italy; ^4^Pasteur Institute, Cenci Bolognetti Foundation, Sapienza University, Rome, Italy; ^5^Department of Internal Medicine and Medical Specialties, Sapienza University, Rome, Italy

**Keywords:** Tangier, hypoalphalipoproteinemia, neuropathy, demyelinating

## Abstract

Tangier disease is an autosomal recessive disorder characterized by severe reduction in high-density lipoprotein cholesterol and peripheral lipid storage. We describe a family with c.5094C > A p.Tyr1698* mutation in the ABCA1 gene, clinically characterized by syringomyelic-like anesthesia, demyelinating multineuropathy, and reduction in intraepidermal small fibers innervation. In the proband patient, cardiac involvement determined a myocardial infarction; lipid storage was demonstrated in gut, cornea, and aortic wall. The reported ABCA1 mutation has never been described before in a Tangier family.

## Introduction

Tangier disease (TD) is a rare disorder of high-density lipoprotein (HDL) metabolism with very low levels of HDL cholesterol (HDL-C) and apolipoprotein A-I (Apo A-I) and by the accumulation of cholesteryl esters (CEs) within macrophage-rich tissues. TD is caused by loss-of-function (LOF) mutations in the ATP-binding cassette transporter A1 (*ABCA1*) gene (1–3), encoding the membrane transporter ABCA1. This transporter plays an important role in the efflux of free cholesterol (FC) from peripheral cells and its transfer to lipid-poor Apo A-I particles, which represents the first stage of reverse cholesterol transport (RCT) pathway (4–8). Therefore, ABCA1 deficiency leads to intracellular accumulation of CE, precludes the conversion of the lipid-poor Apo A-I particles into pre-β HDL, and promotes a rapid catabolism of the poorly lipidated Apo A-I, mostly by the kidney (9). As a consequence, abnormal plasma HDL particles, enlarged orange–yellow tonsils, anemia, thrombocytopenia, peripheral neuropathy, and corneal opacifications are the typical clinical features of TD patients (10), although presenting with a high degree of variability even in the same family (11). TD is often, but not always, associated with an increased risk of coronary artery disease (CAD) (10). Nervous system involvement in TD was already documented in 70s and 80s by Dyck et al. (12) and Pietrini et al. (13, 14). Rare is the presentation of syringomyelia-like symptoms. Even rarer is a demyelinating neuropathy (15–18) and for the majority of the described cases genetic data are not available.

Tangier disease is inherited as an autosomal recessive disorder, and affected patients are expected to have LOF mutations in both alleles of *ABCA1* gene. Heterozygous relatives of TD patients may present an intermediate phenotype of low HDL C and approximately 50% reduction in ABCA1-mediated cell cholesterol efflux (19). The human *ABCA1* gene on chromosome 9q22-q31 contains 50 exons and spans 150 kb. At present, over 200 *ABCA1* mutations^1^ have been identified in TD patients. They have been reported throughout the gene, particularly in exons encoding the extracellular domain and the ATP-binding cassettes (nucleotide binding folds) of the 2261 amino acid protein. Two-third of mutations listed in the database are missense mutations while the others are nonsense or frameshift mutations. Conversely, those affecting intronic sites have been reported only in few cases (20).

In the present article, we describe a novel *ABCA1* mutation in a family with TD associated with an infrequent neurological presentation.

### Index Patient

A 55-year-old Caucasian female, AC, living in a small town near to Matera, presented in October 2013 to Neuromuscular Disease Center in Umberto I Hospital, Sapienza University of Rome, with a reduction of strength in lower limbs. AC evidenced an inexhaustible lateral nystagmus, symmetric facial hypoesthesia to pain, bilateral Bell’s phenomenon with upper and lower facial hyposthenia, left eyelid myokymia, distal deficit of strength in upper limbs, prevailing in the right arm, mild reduction of strength in all muscles of lower limbs [4/5 medical research council (MRC)], more evident in right dorsal and plantar foot flexors (3/5 MRC), and atrophy in intrinsic hands muscles. Suspended bilateral hypoesthesia to pain C3-D12 and postural bilateral low amplitude hand tremor were also evidenced.

Her medical history was significant for tonsillectomy at age 5 and adenoidectomy at age 7 for not specified reasons. In 2012, she underwent blepharoplasty for bilateral lagophthalmos. In 2013, she had a myocardial infarction, treated with double by pass and mitral valve replacement with biologic Carpentier valve. In the same year, she underwent surgical removal of seven colon polyps. In 2002, she was presented with distal symmetrical paresthesia in upper limbs; in 2005, she underwent surgery for bilateral carpal syndrome. In that circumstance, an axonal motor neuropathy in lower limbs was found. In the next 2 years, she noted a progressive hypotrophy in the intrinsic muscles of the hands, with distal to proximal reduction of strength. A few months later, a bilateral sensorineural hearing loss was diagnosed.

AC was admitted in June 2014 for further investigation. Cerebral, brainstem, and medullar MRI was effected to rule out vascular encephalopathy and syringomyelic cavity. Abdomen ultrasound (US) was performed to seek a visceromegaly, as described in the literature. She underwent echocardiogram to investigate the effects of myocardial infarction. A peripheral blood smear was sent for finding stomatocytes. She underwent autonomic function tests to rule out an involvement of the cardiac autonomic system. It was required an eye examination to search for corneal opacities. Muscle biopsy was performed for histopathological characterization and skin punch biopsy for the study of small fibers. Biopsies were taken after local anesthesia using a 3-mm disposable. Three sections randomly chosen from each biopsy were immunoassayed with polyclonal anti-protein-gene-product 9.5 (PGP 9.5) antibodies using the free-floating protocol for bright field immunohistochemistry. The linear epidermal nerve fiber (ENF) density was calculated according to the guidelines given by the European Federation of Neurological Societies (21).

**Figure 1:**
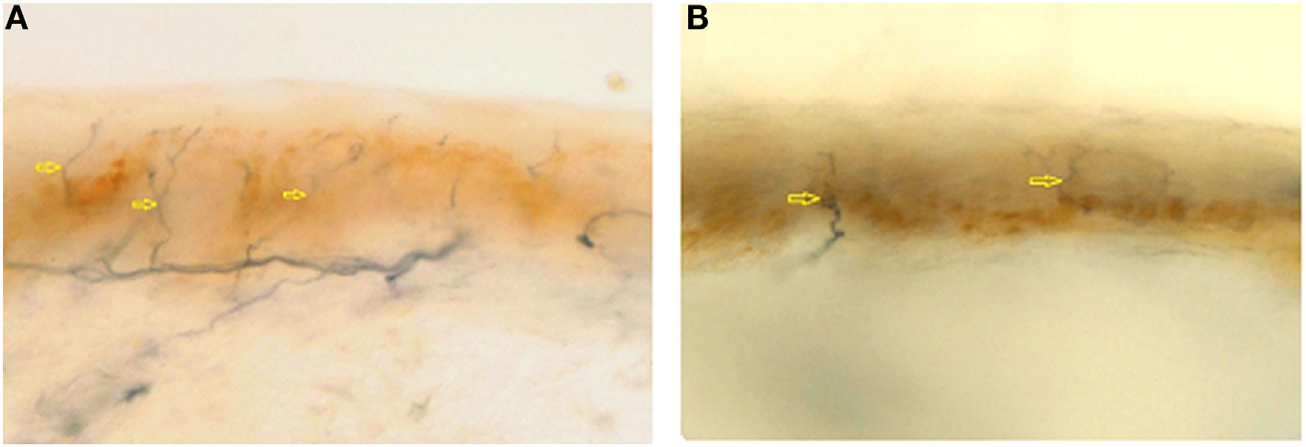
**Epidermal nerve fibre density from first trigeminal branch in a normal subject (A) (normal value: 23.0 ± 6.7) and in AC (B) (1.6/mm)**. Arrows indicates nerve fibers.

Cerebral, brainstem, and medullar MRI showed only a chronic vascular encephalopathy. Abdomen US evidenced a finely compact liver echostructure, angiomyolipoma in the left kidney, and aortic intimal medial increase in thickness with atheroma, without visceromegaly. Echocardiogram evidenced a reduced global systolic function, with 44% ejection fraction, mild pulmonary hypertension (estimated PAPs 37 mmHg), and initial signs of dilated cardiomyopathy, as demonstrated by tetravalvular insufficiency. Peripheral blood smear showed red blood cell anisopoikilocytosis. Tilt test evidenced basal hypotension with adequate peripheral vascular sympathetic response and probably iatrogenic marked reduction in heart rate variability (patient is on beta-blockers following myocardial infarction). Eye examination showed two corneal leucomas in the right eye; a corneal disepithelization was demonstrated by fluorangiography. Muscle biopsy presented fiber II hypotrophy. Skin biopsy, performed in the territory of distribution of first trigeminal branch, D5 dermatome and thigh, showed a wide reduction in intraepidermal innervations (Figure 1), 1.6, 0, and 4.3/mm, respectively, thus explaining the pseudosyringomyelic pattern found.

### Family Study

**Figure 2:**
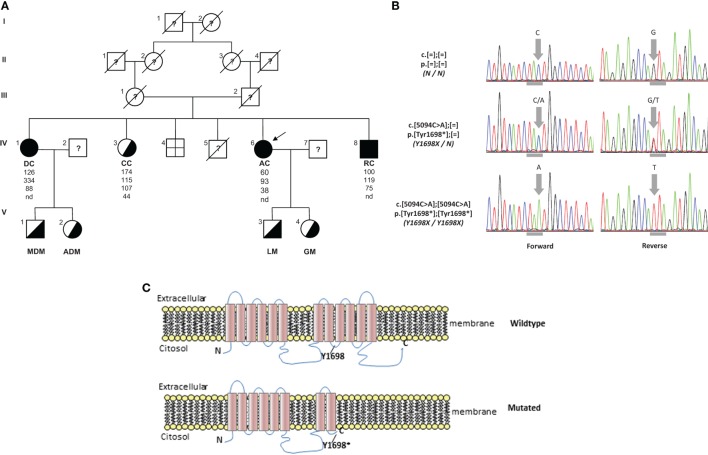
**(A)** Pedigree of TD family. Squares indicate male family members and circles female family members. Slashes indicate deceased people and arrows indicate proband. Roman numerals to the left of the pedigree indicate the generation; numerals to the upper left of each symbol indicate the individual family member. Filled symbols indicate homozygotes and half-filled symbols heterozygotes for the Y1698X mutation in the ABCA1 gene. The columns under each symbol indicate, from top to bottom, the total cholesterol, triglycerides, LDL, and HDL-C concentration (mg/dl). **(B)** Electropherograms reporting nucleotide change in carriers of Y1698X mutation in the ABCA1 gene. Horizontal gray marks under electropherograms indicate the reading frame of codon interested by the mutation. **(C)** Wild type and prediction of Y1698X mutation on the structure of ABCA1 protein. nd, not detectable.

AC family tree is shown in Figure 2A. Her parents were first-degree cousins. She is the fifth of six siblings. One brother was born dead; one brother was dead 2 days after his birth for pneumonia. The oldest sister, DC, is 66 years old and reported a clinical history a myocardial infarction at age of 55 years, aortic and mitral valve replacement, essential hypertension, and hands osteoarticular deformities. The second sister, CC, is 64 years old and presents a non-specified cardiac valve replacement and lower limbs paresthesia. The brother, RC, is 52 years old and presents mild-moderate mitral valve insufficiency, mild tricuspid valve insufficiency, and severe hypoesthesia for pain in his upper limbs (he burned his arm without feeling pain). DC presents clinical findings similar to AC. CC has deficit of strength only in orbicularis oris and hypoesthesia for temperature in her upper limbs. RC presents reduction of strength in orbicularis oris, left foot-drop, and hypoesthesia for pain in upper limbs.

### Electrophysiological Findings

AC presented a definite demyelinating neuropathy, as defined by EFNS criteria (22), with secondary axonal loss. Amplitude, latencies, and conduction velocities of different explored nerves are summarized in Table 1. Distal right ulnar cMAP presented a temporal dispersion and a slow conduction velocity in the under elbow–wrist and above elbow–under elbow tract, with absence of *F* wave, and presence of multiple *A* waves. *F* wave from right median was absent. Left median presented temporal dispersion in elbow–wrist and ERB point–elbow tracts. EMG showed neurogenic potentials and deficit in spatial recruitment in right extensor carpi, bilateral orbicularis oris, and right first digital interosseous (FDI), where fibrillation potentials were detected. She also underwent LASER evoked potentials in order to investigate small fibers: normal from right thigh, no signal from right D5 (LASER anesthesia) and right V1.

**Table 1:** **Electrophysiological findings in TD family members**.

	DC		CC		AC		RC		Control values
	R	L	R	L	R	L	R	L	
**SNC**									
SCV (m/s)
Sural	Abs	Abs	53.8	50.0	52.4	50.0	48.0	45.0	>39.5
Ulnar	Abs	Abs	50.0	50.0	Abs	54.5	50.0	55.0	>47.0
Median	–	–	57.5	60.0	Abs	44.4	48.0	50.0	>49.2
Radial	–	–	60.0	66.0	Abs	26.0	60.0	75.0	>45.0
SNAP (μV)									
Sural	Abs	Abs	28.4	21.4	12.6	8.3	2.4	7.1	>6.3
Ulnar	Abs	Abs	8.4	10.9	Abs	3.6	4.8	5.6	>7.5
Median	–	–	14.6	11.2	Abs	2.8	9.8	8.2	>15.8
Radial	–	–	49.9	54.0	Abs	13.7	7.1	20.1	>23.5
**MNC**									
DML (ms)									
Medial plantar	5.3	5.7	3.2	3.7	3.9	4.2	3.9	5.1	<4.8
Peroneal	–	–	4.4	3.2	3.2	2.9	4.2	5.6	<4.5
Ulnar	4.5	4.8	2.8	2.8	5.7	1.8	2.4	2.3	<2.8
Median	–	–	2.4	3.9	6.1	4.2	4.2	3.7	<3.2
Facial	3.0	3.1	2.9	2.9	2.4	2.9	2.7	2.9	<2.1
MCV (m/s)									
Medial plantar	n.d.	n.d.	47.1	54.0	45.7	50.0	45.0	47.0	>40.3
Peroneal	–	–	51.0	45.5	47.9	44.9	40.0	39.0	>42.5
Ulnar	26.1	25.2	59.0	61.0	30.0	43.5	55.0	50.0	>50.4
Median	–	–	62.0	56.8	27.2	43.1	40.0	39.0	>51.4
Distal/proximal cMAP (mV)									
Medial plantar (A/K)	0.1/n.d.	0.3/n.d.	6.4/4.9	8.6/7.7	5.7/4.7	7.4/4.9	13.1/7.8	12.5/7.8	>8.7
Peroneal (A/UCF/ACF)	–	–	9.7/9.7/9.2	7.8/5.8/5.6	6.1/4.6/5.0	6.1/5.1/4.7	3.5/3.5/3.5	1.2/1.1/1.1	>3.4
Ulnar (W/UE/AE/EP)	3.4/2.2/–/–	2.3/1.5/–/–	16.6/16.6/13.2/12.8	12.5/12.6/12.0/12.0	0.4/0.3/0.3/0.3	6.6/6.6/6.6/4.3	11.1/9.0/7.4/7.4	7.8/7.4/7.2/7.2	>8.3
Median (W/E/EP)	–	–	14.3/14.5/13.4	6.1/6.0/6.0	0.3/0.3/0.3	1.8/1.8/1.8	5.3/4.5/4.3	4.2/3.3/3.3	>9.0
Facial	0.1	0.2	1.8	2.0	0.8	0.5	0.5	0.8	>1.8

*R, right; L, left, –, not done, n.d., not detectable; Abs, Absent; SNC, sensory nerve conduction; SCV, sensory conduction velocity; SNAP, sensory nerve action potential; MNC, motor nerve conduction; DML, distal motor latency; MCV, motor conduction velocity; Cmap, compound motor action potential; W, wrist; UE, under the elbow; AE, above the elbow; E, elbow, EP, Erb point; A, ankle; K, knee; UCF, under the caput fibulae; ACF, above the caput fibulae*.

DC showed absence of SNAP from bilateral sural and ulnar nerves and low cMAP amplitude in bilateral plantar medial and ulnar at motor ENG exam.

Moreover, ulnar nerves presented temporal dispersion in the under elbow–wrist tract and a slow conduction velocity with delay in *F* waves. EMG showed neurogenic potentials and deficit in spatial recruitment in right anterior tibial, bilateral FDI, and bilateral orbicularis oris.

CC presented normal SNAP, cMAP, and NCV from examined nerves. EMG showed neurogenic potentials in bilateral FDI.

RC presented a mild diffuse reduction of sensory and motor nerves amplitude of explored nerves, with a temporal dispersion in the under caput fibulae–ankle tract and absence of *F*-wave in left deep peroneal. EMG showed neurogenic potentials from tibialis anterior bilaterally, right medial gastrocnemius, left vastus lateralis, and right FDI. He also underwent LASER evoked potentials in order to investigate small fibers: normal from right thigh, increase in cortical latency from right D10 dermatome; no cortical signal from right D8 (LASER anesthesia).

### Skin Biopsy

Skin biopsy was performed in DC from proximal and distal lower limb and in CC from proximal and distal upper limb. DC presented a wide reduction in intraepidermal innervations (6.3/mm from proximal site; 0.3/mm from distal site). CC presented normal intraepidermal innervations (12.4/mm from proximal site; 10.9/mm from distal site).

### Laboratory Assessment

AC, DC, and RC presented almost undetectable HDL cholesterol (HDL-C) and extremely low Apolipoprotein A1 levels in their plasma (Table 2). CC, though not presenting very low HDL-C, had low Apolipoprotein A1 levels. AC had low total and LDL cholesterol (LDL-C), but she was in therapy with statin. DC also presented hypertriglyceridemia. Other blood tests were normal.

**Table 2:** **Plasma lipid values in TD family members**.

	DC	AC	CC	RC	Reference values
Genotype	Hom	Hom	Het	Hom	
Total cholesterol (mg/dl)	126	60	174	100	145–199
HDL (mg/dl)	n.d.	n.d.	44	n.d.	>45
LDL (mg/dl)	88	38	107	75	<129
Triglycerides (mg/dl)	334	93	115	119	40–200
Apolipoprotein A1 (mg/dl)	n.d.	24	115	n.d.	>129
Apolipoprotein B (mg/dl)	121	64	100	104	60–117

*n.d., not detectable; Hom, homozygous; Het, heterozygous*.

The clinical history, together with objective and electrophysiological findings and plasma lipid assessment, suggested diagnosis of TD. Therefore, a genetic test was requested.

### Mutational Analysis of ABCA1 Gene

We resequenced the *ABCA1* gene; genomic DNA was extracted from peripheral blood leukocytes by the QIAamp Blood DNA kit (Qiagen, Hilden, Germany). The promoter, 50 exons and exon–intron junctions of the *ABCA1* gene were PCR-amplified and sequenced by primers as reported (23) PCR reactions were performed in a total volume of 15 μl with 50 ng of genomic DNA, 6 pmols of each primer, 0.25 units of GoTaq hot start polymerase (Promega, Fitchburg, WI, USA), 175 μM of each dNTP (Fermentas, Waltham, MA, USA), 1.5 mM MgCl2 and 1× manufacturer’s buffer. The PCR cycle was the following: 2′ 95°C; 35 cycles of 45″ 94°C, 1′ 30″ 58°C, 2′ 30″ 72°C followed by 7′ 72°C. PCR products were purified by a common protocol based on thermosensitive alkaline phosphatase (Fermentas, Waltham, MA, USA) and exonuclease (USB Corporation, Cleveland, OH, USA). Sequencing reactions were performed by using the Big Dye Terminator Reaction Kit version 1.1 (Applied Biosystems, Foster City, CA, USA) according to the manufacturer’s instructions. Sequencing products were purified by Montage SEQ96 sequencing reaction cleanup kit (Millipore, Billerica, MA, USA) following manufacturer’s instructions and subsequently analyzed by the genetic analyzer ABI PRISM 3130*xl* (Applied Biosystems, Foster City, CA, USA). The sequence data were analyzed by using the software SeqScape (Applied Biosystems, Foster City, CA, USA) personalized for the semiautomatic recognition of sequence variations possibly found in the studied zones.

For the analysis of human ABCA1 cDNA and protein, the GenBank NM_005502.3 and NP_005493.2 were respectively used. The *in silico* prediction of the effect of functionally uncharacterized sequence variations found in the proband was performed, depending on the kind of sequence variation, using the following software: PolyPhen-2,^2^ SIFT,^3^ automated splice site analysis,^4^ NetGene2,^5^ GeneMark,^6^ TESS,^7^ TFBIND,^8^ and TFsearch.^9^ In particular, the *in silico* prediction of truncated protein was obtained by the ExPASY Translate tool.^10^

The novel mutation found was labeled according to the Human Genome Variation Society (DNA level; protein level)^11^.

The resequencing of *ABCA1* in the proband evidenced the mutation c.5094C > A p.Tyr1698* (legacy name Y1698X) (Figure 2B). It was a novel mutation introducing a stop codon in the exon 37 of *ABCA1* gene. It was expected to generate a truncated protein, missing the last four intramembrane repeats (Figure 2C). This mutation was found in homozygosity in DC e RC and in heterozygosity in CC (Figure 2A). In addition, this mutation was also found in heterozygosity in the two sons of DC and AC. In the proband, also several polymorphisms and functionally uncharacterized sequence variations were found. The *in silico* analysis of these variations evidenced that they were benign.

## Discussion

In this report, we describe a family characterized by a familial HDL deficiency (FHD), with a novel homozygous nonsense mutation in the *ABCA1* gene. Mutation analysis predicted the synthesis of a truncated protein (Figure 2C). Neurological symptoms represented the prevalent clinical sign in the proband. It has been reported that several different inherited defects involving the apolipoprotein (APO)AI, ABCA1, or lecithin:cholesterol acyl transferas (LCAT) genes may be responsible for FHD (24). Also APOA1-related amyloidosis is characterized by a small fiber neuropathy. However, the presence of neurological symptoms associated with history of tonsillectomy and myocardial infarction, but not of kidney function impairment (which is typical of LCAT-associated FHD) nor cardiomyopathy [which is typical of APOA1-related amyloidosis – (25)] should point toward the diagnosis of ABCA1-associated TD.

The clinical phenotype of AC was peculiar being characterized by a syringomyelic-like anesthesia with electrophysiological findings of a demyelinating neuropathy with secondary axonal loss and small fibers involvement. In DC, AC, and RC both pseudosyringomyelic and demyelinating patterns were found. Concerning the pseudosyringomyelic pattern, spinal MRI did not show any medullar cavities in AC, as described by other Authors (14). Schmalbruch et al. (26) demonstrated loss of small spinal ganglion cells in light and electron microscopy. In our patients, LEP and skin biopsy confirmed this data, showing a global reduction in ENF density; in AC, no small fiber was found in dorsal dermatome (D5), thus partially explaining the pseudosyringomyelic pattern. It has been proposed that lipid accumulation and fiber loss may be detected in peripheral nerves, but much more important in dorsal roots and sensory nerves, assuming the form of a primary neuronopathy with secondary axonal degeneration (27–30). As proposed by Zuchner et al. (28), this phenomenon could not be ascribed to impaired lipid efflux from Schwann cell and attributed to other ABCA1 substrates, thus explaining mutation-specific differences.

**Figure 3:**
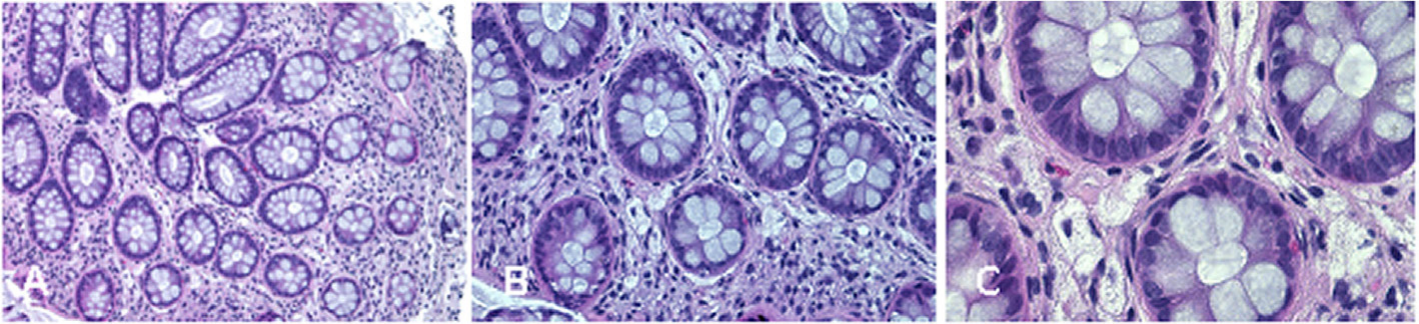
**Colon biopsy with foamy histiocytes (A) 10×, (B) 20×, and (C) 40×**.

Complications of peripheral neurological system are a rather common feature of TD (13). To date, seven different mutations in *ABCA1* have been characterized in neuropathic or pseudosyringomielic cases of TD (28, 30, 31). They have been located in different regions of the gene, but have in common to leading to premature termination of ABCA1 translation and the nearly total absence of ABCA1 protein. It is noteworthy that also the novel variant reported in our family is a nonsense truncating mutation, even though it is located on exon 37, thus expecting to generate a longer truncated protein, missing only the last four intramembrane domains (32). Truncated protein might not be expressed on cytoplasmic membrane and be discarded in the endoplasmic reticulum. Unfortunately, we did not directly evaluate the functional effect of this mutation. Nevertheless, the clinical phenotype of affected individuals is strongly suggestive for a severe impairment of ABCA1-mediated cholesterol removal from cells. Indeed, we found in the proband corneal leucomas, kidney angiomyolipoma, and aortic intimal medial increase in thickness, with atheroma. Colon biopsy performed before hospitalization showed foamy histiocytes (Figure 3), as previously described in a Turkish family (33). Unfortunately, AC underwent a tonsillectomy when she was young and no data were available about her tonsils. All together, these findings strongly suggest that TD patients carrying severely disruptive mutations affecting cholesterol removal may be more prone to develop neuropathy. However, we cannot exclude that the neurological manifestations of TD relies not only on the *ABCA1* mutation type and location, but also on not yet identified genetic and environmental factors, as it has been demonstrated for the TD-related atherosclerotic damage (34).

Physiopathological explanations of demyelinating features in TD remain unclear. Recent evidence suggests paranodal malfunction due to cholesterol ester accumulation in Schwann cells and tomacula formations (29). Abnormal lipid storage was also detected in the Schwann cells of unmyelinated fibers (29). Other reports investigating for lipid storage in CNS (35, 36) and in muscle (37) did not provide any evidence of it.

Unfortunately, no therapy is available for TD. In one patient with established coronary heart disease, none of used lipid lowering agents (niacin, gemfibrozil, estrogen, or lovastatin) elevated HDL above 5 mg/dl (38). However, several attempts are under way to develop new treatments. One of most promising is a human apoA-I-containing HDL-mimetic particle (CER-001). It has been recently shown (39) that CER-001 determined a significant increase of apoA-I and HDL-C levels in *in vitro* cellular cholesterol efflux. Moreover, carotid mean vessel wall area significantly decreased compared with baseline.

## Conclusions

Other authors (15, 16) described TD families with demyelinating multineuropathy or syringomyelic-like syndrome. However, the concomitant presentation of these two neurological symptoms is uncommon. Our findings support the notion that a lipid profile should be included in the screening of demyelinating polyneuropathies in order to improve diagnosis, thus avoiding inappropriate treatments. We suggest that suspended anesthesia could be explained by a focal small fibers depletion, as demonstrated by LEP and skin biopsy. Further studies are necessary to confirm genotype–phenotype relationship and to investigate pathophysiological mechanisms of demyelination and focal loss of small fibers.

## Compliance with Ethics Guidelines

This article does not contain any studies with human or animal subjects performed by the any of the authors. Additional informed consent was obtained from all patients for which identifying information is included in this article.

## Author Contributions

MC: writing paper, electrophysiological studies, and management of patient during the hospitalization. CC, VF, and EO: electrophysiological studies. AB: skin biopsy and LASER evoked potentials, writing materials and methods for skin biopsy. CG: muscle biopsy. SB, GT, and ML: genetic analysis, evaluation of lipid assessment and writing materials and methods and results for genetic analysis, and data interpretation. MA: writing paper, genetic analysis, evaluation of lipid assessment and writing materials and methods and results for genetic analysis, and data interpretation. MI: clinical evaluation, electrophysiological studies, data interpretation, and writing paper.

## Conflict of Interest Statement

The authors declare that the research was conducted in the absence of any commercial or financial relationships that could be construed as a potential conflict of interest.

## Footnotes

^1^http://www.hgmd.cf.ac.uk/ac/gene.php?gene=ABCA1.

^2^http://genetics.bwh.harvard.edu/pph2/.

^3^http://sift.jcvi.org/.

^4^https://splice.uwo.ca.

^5^http://www.cbs.dtu.dk/services/NetGene2.

^6^http://exon.biology.gatech.edu/.

^7^http://www.cbil.upenn.edu/tess/.

^8^http://tfbind.hgc.jp/.

^9^http://diyhpl.us/~bryan/irc/protocol-online/protocol-cache/TFSEARCH.html.

^10^http://web.expasy.org/translate/.

^11^http://varnomen.hgvs.org/.
